# The Role of Specific Warm-up during Bench Press and Squat Exercises: A Novel Approach

**DOI:** 10.3390/ijerph17186882

**Published:** 2020-09-22

**Authors:** Bruno Ribeiro, Ana Pereira, Pedro P. Neves, António C. Sousa, Ricardo Ferraz, Mário C. Marques, Daniel A. Marinho, Henrique P. Neiva

**Affiliations:** 1 Department of Sport Sciences, University of Beira Interior, 6201-001 Covilhã, Portugal; ribeiro.aikido@gmail.com (B.R.); pedroneves93@hotmail.com (P.P.N.); antonio_carlossousa@hotmail.com (A.C.S.); rmpf@ubi.pt (R.F.); mariomarques@mariomarques.com (M.C.M.); dmarinho@ubi.pt (D.A.M.); 2 Research Center in Sports Sciences, Health Sciences and Human Development, CIDESD, 6201-001 Covilhã, Portugal; 3 Department of Science and Technology, Polytechnic Institute of Setubal, 2910-761 Setúbal, Portugal; anapereiraphd@gmail.com

**Keywords:** pre-exercise, strength, velocity, power, work

## Abstract

The current study aims to verify the effects of three specific warm-ups on squat and bench press resistance training. Forty resistance-trained males (19–30 years) performed 3 × 6 repetitions with 80% of maximal dynamic strength (designated as training load) after one of the following warm-ups (48 h between): (i) 2 × 6 repetitions with 40% and 80% of the training load (WU), (ii) 6 × 80% of training load (WU80), or (iii) 6 × 40% of the training load (WU40). Mean propulsive velocity (MPV), velocity loss (VL), peak velocity (PV), time to achieve PV, power, work, heart rates, and ratings of perceived exertion were analyzed. In squat exercises, higher MPV were found in WU80 compared with WU40 (2nd set: 0.69 ± 0.09 vs. 0.67 ± 0.06 m.s^−1^, *p* = 0.02, ES = 0.80; 3rd set: 0.68 ± 0.09 vs. 0.66 ± 0.07 m.s^−1^, *p* = 0.05, ES = 0.51). In bench press exercises, time to PV was lower in WU compared with WU40 (1st set: 574.77 ± 233.46 vs. 694.50 ± 211.71 m.s^−1^, *p* < 0.01, ES = 0.69; 2nd set: 533.19 ± 272.22 vs. 662.31 ± 257.51 m.s^−1^, *p* = 0.04, ES = 0.43) and total work was higher (4749.90 ± 1312.99 vs. 4631.80 ± 1355.01 j, *p* = 0.01, ES = 0.54). The results showed that force outputs were mainly optimized by WU80 in squat training and by WU in bench press training. Moreover, warming-up with few repetitions and low loads is not enough to optimize squat and bench press performances.

## 1. Introduction

Warm-up has been identified as essential to maximizing the athlete’s performance in different sports and physical activities [1]. The activities performed before the main competition event or training session seem to increase body temperature and, in this way, cause the athlete to benefit from decreased stiffness, increased nerve conduction rate, and increased metabolic efficiency [2,3]. Based on this, over the years, the benefits of warm-up were taken for granted, and it became usual practice, sometimes without enough scientific evidence to support it. Warm-up has been shown to improve individual (e.g., cycling, running, swimming) and team sports (football, rugby) [4,5,6], but some contradictory results have highlighted that specific warm-up designs (type, intensity, volume) should be further understood. Moreover, little is known regarding the effect of different warm-ups in some other specific activities, such as resistance training [7,8,9]. Each athlete is used to warming-up before any resistance training in order to obtain higher performance levels in every single session [8,9]. However, improper use of volume and/or intensity of the tasks during the warm-up can compromise the following performance, as shown in other sports [10].

Previous research has found an increase of 3% of upper body power during medicine ball throws after specific warm-ups in bench press exercises with the maximal external load that each subject can move properly for 5 repetitions, contrary to higher or lower loads [11]. The number of repetitions performed until fatigue in bench press and leg extension exercises seemed to be largely influenced by the specific warm-up intensity [7]. When a specific warm-up was performed at loads close to the maximum (90% of maximal dynamic [1RM] load), the results demonstrated that the ability to produce maximal dynamic strength could be positively affected [7]. It seems that there is a tendency to use high external loads before power exercises, using the underlying mechanisms resulting from postactivation potentiation, but little are known about the intensities before resistance training sessions [12]. In fact, the postactivation potentiation has been suggested to optimize maximal strength [12,13], but only recently has it been applied to multiple resistance training sets [9]. It was found that adding three repetitions performed with 90% 1RM load to a specific warm-up of 8 repetitions with 50% 1RM caused an increase of work and number of repetitions performed during three sets until failure with 75% 1RM in bench press exercises [9]. The authors suggested that this acute optimization of training could potentially increase results over the long term.

Strength performance can be improved using a specific warm-up [7,14]; nevertheless, the optimal load to be used remains uncertain. The efficiency of warm-up practices depends on the balance between fatigue onset and muscle potentiation [15]. This balance is provided by several factors such as training experience [15], the rest period between warm-up sets and the main activity [16], and the volume and intensity performed [17]. To our knowledge, the few existing studies on the influence of specific warm-up loads in resistance exercise should be deepened by analyzing other variables. Most of the studies used the determination of 1RM load or multiple submaximal repetitions as dependent variables, disregarding the daily variation of 1RM performance [7,18,19]. Furthermore, a progressive increase of external loads is needed to evaluate the 1RM load, and this increases the confounding factors, perhaps reducing the effects of prior warm-up and increasing the fatigue effect over the assessment. This compromises any results and does not allow the researchers to better understand the effects of different warm-ups on force and power production. The novelty of the current study would be the evaluation of the resistance exercise performance using mechanical variables such as the velocity of movement, which can be tested in a real resistance training set with submaximal external loads [20,21]. Linear position technology has been used to measure velocity, displacement, power, force variables to determine and monitor performance in resistance exercises [20,21,22]. Therefore, controlling the movement velocity at which a given resistance exercise is executed is critical to ensure that training is as effective as possible and will allow a reliable analysis of the effect of previous warm-up procedures.

Knowing the importance of warm-ups for strength optimization and the need to avoid the effects of muscle fatigue, it is important to observe the effects and influence of using different specific warm-up designs to increase the efficiency of resistance training exercise performance. For instance, during resistance training, an appropriate warm-up would increase force development against the same training external load, improving the training efficiency, training intensity, and, therefore, long-term adaptations to develop higher force values and/or higher velocities [20,21,22,23]. Therefore, the purpose of this study is to analyze the effects of different specific warm-ups on bench press and squat exercises, evaluating mechanical responses (propulsive velocity and mechanical power) and physiological (heart rate) and psychophysiological (ratings of perceived exertion: RPE) variables during a typical resistance training set. It was hypothesized that a specific warm-up, with increasing intensities, would optimize resistance training by increasing propulsive velocity and mechanical power produced in bench press and squat exercises.

## 2. Materials and Methods

### 2.1. Experimental Approach to the Problem

The current study tried to further understand the importance of warm-up intensities for resistance training exercises performance and then to realize the optimal warm-up load that should be used pretraining. The effects of the warm-up load in resistance training performance were evaluated by analyzing the bar movement velocity using a recent validated device [21,24,25,26]. We know that increasing the movement velocity against a load will result in greater performance; this will probably influence the acute and long-term training effects [20,22,24]. One of the novelties of the current study is the assessment of the resistance performance using mechanical variables such as the velocity of the movement, which could be the best reference for measuring the real effort of the athlete with submaximal external loads [20,21].

A crossover research design was used to analyze the effects of a warm-up on mechanical responses, mean propulsive velocity (MPV), propulsive mechanical power (MPP), and physiological (heart rate) and psychophysiological variables (ratings of perceived exertion: RPE). After the adaptation session, four evaluation sessions were performed on four different days (48 h in between). The first evaluation was to determine the 1RM load of each participant in squat or bench press exercise. Then, each participant performed resistance training (3 sets of 6 repetitions at 80% 1RM loads) in three subsequent sessions. These resistance exercises were chosen because of their relevance in resistance training, thus commonly used by conditioning specialists and coaches for sports training. Moreover, the intensity of 80% 1RM is widely used in traditional resistance training, and it is in the optimal range of relative intensities (30–80% 1RM) that have been suggested to improve long term muscular performance [27,28,29]. This resistance training was performed randomly after three different warm-ups: the progressive-intensity (WU) warm-up that included 2 sets of 6 repetitions, with 40% and 80% of the training load; the warm-up that included 1 set of 6 repetitions, with 80% of training load (WU80); the warm-up that included 1 set of 6 repetitions, with 40% of training load (WU40).

### 2.2. Subjects

Forty men aged 19–30 years old volunteered to participate in the current study. From these, 14 men (24.43 ± 3.48 years; 1.76 ± 0.71 m in height; 77.71 ± 10.35 kg of body mass) were evaluated using squat exercises, and 26 men (22.19 ± 1.67 years, 1.77 ± 0.06 m in height, 72.23 ± 8.21 kg of body mass) were assessed using bench-press exercises. Subjects were physically active, engaged in physical activity regularly, with an experience of resistance training for the last two years. Everyone was asked to report any previous illness, injury, or other physical issues that would hinder their performance. The eligibility criteria were being healthy and injury-free, aged between 18 and 35 years old, and having previous experience with back-squat and bench-press exercises. Criteria of exclusion were the evidence of an orthopedic or medical problem or another self-reported issue that would endanger their health. The participants were informed about the study procedures and written informed consent was signed. The investigation was conducted in accordance with the Declaration of Helsinki and was approved by the University of Beira Interior Research Ethics Committee (under the project d1576, October 2015).

### 2.3. Procedures

The first session was used for body composition evaluation (Seca Instruments, Ltd., Hamburg, Germany) and adaptation to testing conditions, where the subjects performed some repetitions with lower loads in bench press or squat exercises, with some orientation about the correct technique. The second session was used to determine the individual load–velocity relationships and estimated 1RM strength in the squat or bench press exercises using a progressive loading test. Then, the last three sessions were used to evaluate performance during a resistance training session with different specific intensities of warm-ups. All procedures were performed using a Smith machine (Multipower Fitness Line, Peroga, Murcia, Spain), similar to other studies, e.g., [23,25,26]. The Smith machine allows only a vertical displacement of the barbell along a fixed pathway and has a very low friction force between the barbell and the support rails, acting identical to free-weights [25]. Moreover, the bar velocity was measured using a linear transducer sampling at 1000 Hz (T-Force System, Ergotech, Murcia, Spain) connected to a 16-bit analog-to-digital converter (Biopac MP100 Systems, Santa Barbara, CA, USA). The T-force System was interfaced with a personal computer to automatically calculate the relevant kinematic variables of the parameters for every repetition [20,21,25].

In the squat exercise, each subject started from the upright position with the knees and hips fully extended, stance approximately shoulder-width apart, and the barbell resting across the back at the level of the acromion. The eccentric phase was performed in a continuous motion until the top of the thighs was below the horizontal plane, and the concentric phase was made at the maximum velocity to the initial position [20,25]. Trained spotters were present on both sides of the barbell when high loads were lifted to ensure safety. In the bench press exercise, each participant lay supine on a flat bench, with their feet resting on the floor and hands placed on the barbell slightly wider than shoulder-width. They were instructed to lower the bar to the chest, just above the nipples, in a controlled manner, and, after approximately 1.0 s of pause, they started the concentric phase of the movement as fast as possible. The momentary pause at the chest between the eccentric and concentric actions was to minimize the contribution of the rebound effect and allow for more reproducible, consistent measurements [28]. The subjects were not allowed to bounce the bar of their chest or to raise their shoulders or trunk off the bench [21].

To determine individual 1RM loads, the initial load was set at 20 and 30 kg for all participants in the bench press and squat exercises, respectively, and was gradually increased by 10 kg increments. The test ended for each participant when the concentric MPV of 0.4 ms^−1^ in the bench press and 0.6 ms^−1^ in the squat was attained, corresponding to 85% 1RM in both [21,25]. Three attempts were executed for light (<50% 1RM), 2 for medium (50–80% 1RM), and only 1 for the heaviest (>80% 1RM) loads. Interset recoveries were 3 min for the light and medium loads and 5 min for the heaviest loads [21,29]. The 1RM was calculated from the MPV attained during the progressive loading test as follows: (100 × load)/(−5.961 × MPV2) − (50.71 × MPV) + 117 for the squat exercise [25], and (100 × load)/(8.4326 × MPV2) − (73.501 × MPV) + 112.33 for the bench press exercise [21].

Three different warm-up conditions were randomly implemented. WU comprised 6 repetitions with 40% of the training load, followed by 6 repetitions with 80% of the training load (1 min interset interval). The warm-up with high intensity included 1 set of 6 repetitions with 80% of the training load (WU80), and the warm-up with low intensity comprised 1 set of 6 repetitions with 40% of the training load (WU40). The effect of performing warm-ups was assessed during a bench press or squat resistance training session performed 3 min after the warm-up. Resistance training consisted of 3 sets of 6 repetitions with 80% 1RM load, with 3 min of interset recovery. The number of sets and loads intensities were selected because of their common use in resistance training in diverse competitive sports and their effects on muscular development and performance improvement [30,31]. During the execution of exercises, there was a continuous orientation to maintain the execution technique. All the subjects reported no fatigue at the start of each session.

All velocity measures identified in this study corresponded to the propulsive phase of each repetition [20,21,24]. The maximal and minimum values of MPV (mean velocity value from the start of the concentric phase until the acceleration of the bar is lower than gravity) over each set, the relative magnitude of MPV loss (VL) within the set and within the training (calculated as the percent loss in MPV from the fastest to the slowest repetition) [24], the peak velocity (PV: maximum instantaneous velocity value reached during the concentric phase at a given load) [32], and the time to achieve PV in each repetition were considered for further analysis. Moreover, considering the propulsive velocity and the load, other mechanical variables were analyzed from the software output, such as the maximal and minimal values of MPP in each set and the work produced in each set and in the entire training.

The heart rate values were assessed at rest (baseline), 1 min after the warm-up, and immediately after training (1 min). Additionally, the rating of perceived exertion (RPE) values was recorded using a 16-points Borg scale (6–20 rates) [33] immediately after warm-up and after the resistance training.

### 2.4. Statistical Analysis

Standard statistical procedures were selected to calculate means, standard deviations (SDs), and 95% confidence intervals. The normality of all distributions was verified with the Shapiro–Wilk test, and parametric statistical analysis was adopted. The effect of the warm-up procedures was analyzed by an ANOVA for repeated measures, with sphericity checked using Mauchly’s test. Posthoc paired *t*-tests were run to additional investigate the effect of each condition. The effect size was calculated to estimate the variance between conditions (partial eta squared: η_p_^2^), and Cohen’s dz (ES) for within-subject comparisons were calculated using the Excel spreadsheet by Lakens [34]. For partial eta squared (η_p_^2^), the cut-off values were interpreted as 0.01 for small, 0.09 for moderate, and 0.25 for large, and ES values of 0.20, 0.60, 1.20, and 2.00 were considered small, moderate, large, and very large magnitudes, respectively [35]. Statistical treatment was performed using the Statistical Package for Social Sciences (IBM SPSS Statistics for Windows, Version 22.0. Armonk, NY: IBM Corp).

## 3. Results

**Figure 1:**
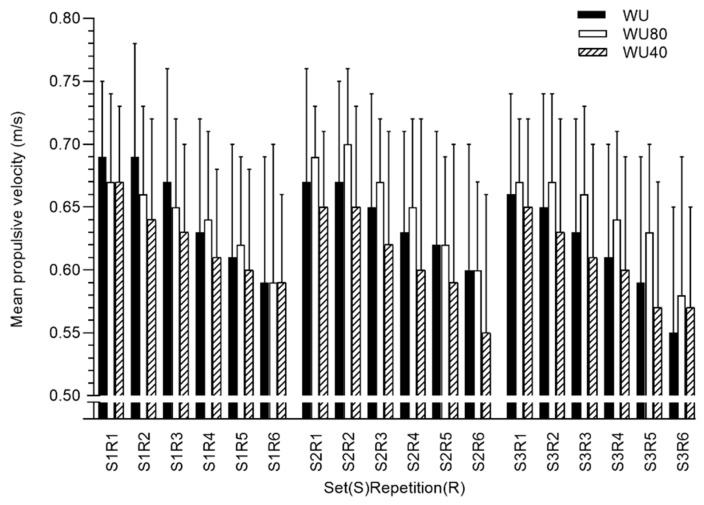
Mean propulsive velocity values in each repetition performed during the squat exercise after WU, WU80, and WU40.

The mean values, differences, and ES for MPVs in the first, second, and third sets in the squat exercise are presented in Table 1. There were small effects in MPV in the first set of the resistance training between warm-up conditions (F = 1.31, *p* = 0.29, η_p_^2^ = 0.09). However, MPV showed to be moderately affected by warm-up conditions in the second (F = 2.78, *p* = 0.08, η_p_^2^ = 0.18) and third sets (F = 2.28, *p* = 0.12, η_p_^2^ = 0.15). In these sets, the participants were able to perform the squat exercises at higher MPV values after WU80, comparing to WU40. The minimal value of MPV in WU40 showed lower values when compared with the other warm-ups during the second set (F = 25.23, *p* < 0.001, η_p_^2^_=_ 0.66). No significant differences were found between conditions in VL during the first (F = 0.29, *p* = 7.48, ηp^2^ = 0.02), second (F = 2.59, *p* = 0.09, ηp^2^ = 0.17) and third sets (F = 0.51, *p* = 0.61, ηp^2^ = 0.04). Curiously, the values were moderately higher in WU40 during the second set. In Figure 1, we observed a tendency for higher MPV values during second and third set after WU80 and lower values after WU40 during second set.

The mean values, differences, and ES for PV and time spent to achieve PV in the first, second, and third sets in the squat exercise are presented in Table 2. The participants were able to attain PV in less time in the second set after WU80 when compared to WU40 (F = 3.09, *p* = 0.06, η_p_^2^ = 0.19).

Table 3 presents the results of the mechanical power and work produced during the squat performance. Despite no differences being found between conditions in MPP in the first (F = 0.81, *p* = 0.46, η_p_^2^ = 0.06), second (F = 1.73, *p* = 0.20, η_p_^2^ = 0.12), and third sets (F = 2.16, *p* = 0.14, η_p_^2^ = 0.14), moderate effects were found with lower values in the WU40 condition. Large effects were found in the minimal values of MPP in the second set (F = 7.73, *p* = 0.02, η_p_^2^ = 0.37), with higher values recorded in WU80 when progressively compared with WU40. No differences were found in the work produced during squat resistance training (F = 0.92, *p* = 0.41, η_p_^2^ = 0.07).

**Figure 2:**
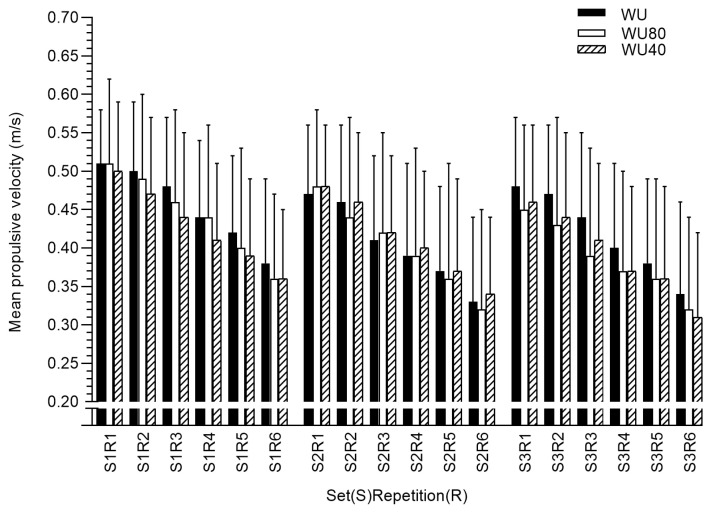
Mean propulsive velocity values in each repetition performed during the bench press exercise after WU, WU80, and WU40.

In bench press exercises, no differences were found between conditions in MPV and VL (Table 4 and Figure 2). However, there were differences in time to PV in first (F = 2.44, *p* = 0.10, η_p_^2^ = 0.09) and second sets (F = 3.11, *p* = 0.05, η_p_^2^ = 0.11) after WU warm-up when compared with WU40 (Table 5).

Table 6 presents the results recorded regarding the mechanical power and work produced during the bench exercise. Despite no differences being found between conditions in MPP, significant results in work can be observed, with progressive warm-ups showing higher values when compared to 40% of training load warm-up in first (F = 3.00, *p* = 0.06, η_p_^2^ = 0.11) and second sets (F = 2.31, *p* = 0.11, η_p_^2^ = 0.08). The total work also presented significant results between these two conditions (F= 2.56, *p* = 0.09, η_p_^2^ = 0.09)

Immediately after performing the three sets of resistance training, no differences were found in heart rate values in the bench press exercise (F = 1.74, *p* = 0.19, np^2^ = 0.06) and in the squat exercise (F = 0.97, *p* = 0.39, np^2^ = 0.07). In the perceived effort after the bench press training, no differences were found between conditions (F = 0.39, *p* = 0.68, np^2^ =0.01), and no differences existed regarding the squat training (F = 2.17, *p* = 0.14, np^2^ = 0.14).

## 4. Discussion

The main purpose of the present study was to analyze the effects of a specific warm-up with different loads on squat and bench press performances, using some novel procedures to measure force outputs. Thus, we compared the force outputs during a typical resistance training set by measuring mechanical variables such as movement velocity and power and physiological and psychophysiological variables. The participants were able to reach higher MPVs in the second and third sets of the squat exercise in WU80 compared to WU40. Nevertheless, in the bench press exercise, the time to PV was lower after the warm-up with progressive intensity. No differences were found in MPP, but there was a tendency for higher work performed after the progressive warm-up. This was highlighted by the higher work values obtained in the bench-press exercise during the first and second sets and overall training compared with WU40. The hypothesis that the propulsive velocity and mechanical power during squat resistance training would be influenced by performing a progressive specific warm-up or 80% of training load was confirmed by the presented results. However, in the bench press exercise, the time to PV, the mechanical power, and work seem to be optimized with a progressive warm-up. The results revealed that each specific warm-up caused different responses according to the following exercise, specifically during the squat and bench press exercise training.

The importance of MPV to monitor resistance training variables such as the intensity and the volume is known, but it can also be considered as the steadiest variable for muscle strength assessment in isoinertial conditions [20,21,24]. Therefore, the effects of warming-up on the velocity of the movement performed during resistance training are fundamental to further understanding warm-up design and procedures and to optimize resistance training and long-term performance. In fact, sports activities require athletes to move, launch, shut, and run at high-speed rates that should be improved by resistance training [4,5], and a warm-up can play an essential role in training optimization. It has been established that specific warm-ups are beneficial to 1RM and the number of repetitions performed with submaximal loads [7,11], but, until now, little was known regarding the quantity and load of the specific warm-up and its influence on resistance training; we, therefore, analyzed the mechanical variables that showed an influence over resistance training performance.

MPV showed a tendency to be higher at the beginning of the squat training session, after WU. However, in the second and third sets, WU80 was the condition with better results. In the bench press exercise, these differences did not exist, perhaps due to the lower quantity of muscles involved and the inferior complexity of the movement [11]. It is expectable that with greater complexity and quantity of muscle involved, the squat exercise could require a more specific warm-up. This led to differences in the first set of squats, with the progressive warm-up giving better results. Moreover, this is evidenced by outputs in the second set, in which WU80 caused higher MPV values and less time to PV. It seemed that after performing just one set of warm-up with 80% of training load (64% 1RM), less fatigue was accumulated comparing with the two sets performed during WU, thus resulting in greater results in the following training sets. Nevertheless, in the bench press exercise, WU was the most effective, resulting in less time to PV in the first sets and a tendency for higher MPV values.

The results of this study corroborate with some investigations that have reported improved strength performance and increased work and number of repetitions after a warm-up with high loads [9]. A specific warm-up can enhance force production after maximum or near-maximal muscle stimulation [7], and some authors have attributed this situation to increased phosphorylation of the myosin regulatory light chain, especially in type II muscle fibers [36]. Moreover, a proper warm-up promotes an increase in body temperature and increased velocity nerve transmission impulses and oxygen availability in the muscular system, decreased connective tissue stiffness, and viscosity of the musculoskeletal system, allowing the body to stay more available for the activity to be performed [1,2,3,37]. In this case, the faster velocities attained and the lower time to achieve maximal velocities could signify better neuromuscular capacity or acute potentiation [24], resulting from a more appropriate stimulus. This was verified in WU and WU80, which were the warm-ups performed with higher loads.

VL and metabolic stress depend considerably on the number of repetitions performed and the functional and structural adaptations in the muscle according to the intensity effort [23]. In the current study, the training VL was similar between warm-ups in both exercises. Nevertheless, the minimal MPV and MPP values were verified after WU40, which supports the lower performances in this condition. The reduction in movement velocity during resistance exercise may be indicative of fatigue [23,25]. On the one hand, the lower values of MPV after WU40 were representative of worse neuromuscular capacity, and, on the other hand, the lower values of minimal MPV and MPP showed decreased strength performance and/or higher levels of fatigue in this warm-up condition. Moreover, regarding the work produced during training, the greatest effects were found after the WU condition, particularly in the bench press exercise, with higher values compared with WU40. The work produced during the resistance training depends on the mass, the vertical displacement of the bar, and the number of repetitions performed during the session. Considering that the external load and number of repetitions were the same between analyzed warm-up conditions, greater work could be caused by some minor changes in acceleration and/or in the displacement of the bar during exercise.

Some limitations should be addressed to the current study. We should be aware that only bench press and squat exercises were analyzed, and cautions should exist when generalizing our findings to other resistance training exercises. Nevertheless, the squat and bench press are some of the most used exercises in strength-related studies and most resistance training contexts. A larger sample would benefit more clear conclusions in some of the analyzed variables, and future studies could also monitor and control the nutritional intake of participants throughout the study, as well as other variables (e.g., hormonal responses and core temperature). Despite these limitations, the results are interesting for all coaches, athletes, and researchers. Most of the studies investigating the effects of warm-ups on muscle force output generally focus on maximal dynamic strength and the maximal number of repetitions, disregarding the confounding effect of the procedures (i.e., the influence of previous repetitions on maximal performance output, adding effects to the warm-up independent variable). Linear position technology, commonly used to measure force outputs and monitor athletic performance, allowed us to measure and analyze the changes in resistance training caused by different specific warm-ups.

## 5. Conclusions

The current study took a novel approach to warm-up research by examining the effects of warm-ups on resistance training using some recent procedures to measure performance. In summary, an increase in force outputs during squat training was found after a specific warm-up, comprising only six repetitions with 80% of the training load. This was clear during the second and third training sets. In the bench press exercise, a progressive-intensity warm-up of two sets of 6 repetitions, with 40% and 80% of training load, was the most effective, resulting in less time to attain maximal velocities and augmented work. In general, the warm-up with 40% of the training load was the one with worse performances in both exercises.

Some practical applications can be drawn. Despite some different responses for each exercise, progressive-intensity specific warm-up and WU80 should be used to maximize performance during resistance training. Specifically, our outcomes allowed us to disclose that higher intensities (i.e., 80% of training load) and only a few repetitions (i.e., 6 repetitions) should be used during specific warm-ups to optimize squat training. However, the bench press exercise should be preceded by more repetitions (i.e., 2 × 6 repetitions) with progressive loads (i.e., 40% and 80% of training load). The present findings can be applied before bench press and squat resistance training but also when assessing maximal dynamic strength. Knowing that the movement velocity can be used to determine maximal dynamic strength and to monitor resistance training performance, our outcomes can provide new insights and recommendations for sports professionals and researchers to improve training efficiency and optimize performance.

## Figures and Tables

**Table 1:** Mean values ± standard deviation of the maximal value of mean propulsive velocity (MPV), minimal MPV values, and velocity loss (VL) in each set and total training (total) in squat exercises. Differences and confidence intervals (95% CI), effect sizes (ES), and *p*-values are also presented.

	WU	WU80	WU40	WU vs. WU80		WU vs. WU40		WU80 vs. WU40	
				Difference (±95% CI)	*p*-Value (ES)	Difference (±95% CI)	*p*-Value (ES)	Difference (±95% CI)	*p*-Value (ES)
MPV [set 1] (m.s^−1^)	0.71 ± 0.08	0.69 ± 0.07	0.68 ± 0.06	0.02 (±0.02)	0.10 (0.48)	0.03 (±0.04)	0.21 (0.40)	0.01 (±0.03)	0.71 (0.15)
MPV [set 2] (m.s^−1^)	0.69 ± 0.09	0.71 ± 0.05	0.67 ± 0.06	−0.01 (±0.04)	0.46 (0.27)	0.02 (±0.04)	0.15 (0.34)	0.04 (±0.03)	0.02 * (0.80)
MPV [set 3] (m.s^−1^)	0.68 ± 0.09	0.69 ± 0.07	0.66 ± 0.07	−0.02 (±0.05)	0.41 (0.13)	0.02 (±0.01)	0.08 (0.58)	0.03 (±0.04)	0.05 * (0.51)
MPVmin [set 1] (m.s^−1^)	0.58 ± 0.10	0.58 ± 0.10	0.58 ± 0.08	0.01 (±0.06)	0.88 (<0.01)	0.01 (±0.04)	0.79 (<0.01)	0.00 (±0.05)	0.98 (<0.01)
MPVmin [set 2] (m.s^−1^)	0.58 ± 0.09	0.59 ± 0.07	0.54 ± 0.11	−0.01 (±0.05)	0.69 (0.12)	0.04 (±0.04)	0.01 ** (0.70)	0.05 (±0.05)	0.04 * (0.60)
MPVmin [set 3] (m.s^−1^)	0.54 ± 0.09	0.57 ± 0.09	0.55 ± 0.09	−0.03 (±0.06)	0.31 (0.34)	−0.01 (±0.04)	0.58 (0.16)	0.02 (±0.06)	0.62 (0.18)
VL [set 1] (%)	17.56 ± 9.14	16.31 ± 10.35	15.11 ± 7.44	1.26 (±8.47)	0.75 (0.09)	2.45 (±6.06)	0.40 (0.23)	1.19 (±5.85)	0.67 (0.12)
VL [set 2] (%)	16.35 ± 5.63	16.32 ± 6.07	20.57 ± 10.89	0.03 (±3.16)	0.99 (0.01)	−4.22 (±4.75)	0.08 (0.51)	−4.25 (±5.66)	0.13 (0.43)
VL [set 3] (%)	19.99 ± 7.11	18.44 ± 9.94	16.86 ± 8.57	1.55 (±6.21)	0.60 (0.14)	3.14 (±5.45)	0.24 (0.33)	1.59 (±8.21)	0.68 (0.11)
VL [total] (%)	26.59 ± 8.58	26.83 ± 10.58	26.09 ± 10.40	−0.24 (±5.40)	0.93 (0.03)	0.50 (±5.53)	0.85 (0.05)	0.74 (±8.33)	0.85 (0.05)

** *p* ≤ 0.01; * *p* ≤ 0.05.

**Table 2:** Mean values ± standard deviation of the peak velocity (PV) and time to reach peak velocity (time to PV) in each set and total training (total) in squat exercises. Differences and confidence intervals (95% CI), effect sizes (ES), and *p*-values are also presented.

	WU	WU80	WU40	WU vs. WU80		WU vs. WU40		WU80 vs. WU40	
				Difference (±95% CI)	*p*-Value (ES)	Difference (±95% CI)	*p*-Value (ES)	Difference (±95% CI)	*p*-Value (ES)
PV [total] (m.s^−1^)	1.33 ± 0.11	1.32 ± 0.07	1.30 ± 0.08	0.01 (±0.05)	0.76 (0.11)	0.03 (±0.05)	0.16 (0.38)	0.03 (±0.04)	0.20 (0.29)
Time to PV [set1] (ms)	586.21 ± 109.87	589.93 ± 133.11	638.00 ± 163.28	−3.71 (±60.23)	0.90 (0.04)	−51.79 (±53.18)	0.06 (0.56)	−48.07 (±50.85)	0.06 (0.55)
Time to PV [set2] (ms)	601.64 ± 110.91	565.86 ± 106.87	623.50 ± 136.80	35.79 (±54.94)	0.18 (0.38)	−21.86 (±45.19)	0.32 (0.28)	−57.64 (±51.25)	0.03 * (0.65)
Time to PV [set3] (ms)	606.93 ± 114.53	582.57 ± 129.29	619.36 ± 191.00	24.36 (±48.06)	0.29 (0.29)	−12.43 (±69.26)	0.71 (0.10)	−36.79 (±76.09)	0.32 (0.28)

* *p* ≤ 0.05.

**Table 3:** Mean values ± standard deviation of the maximal value of mean propulsive power (MPP); 95% CI, effect sizes (ES), and *p*-values are also presented.

	WU	WU80	WU40	WU vs. WU80		WU vs. WU40		WU80 vs. WU40	
				Difference (±95% CI)	*p*-Value (ES)	Difference (±95% CI)	*p*-Value (ES)	Difference (±95% CI)	*p*-Value ES
MPP [set 1] (W)	528.78 ± 107.18	519.29 ± 90.75	510.86 ± 101.92	9.49 (±25.06)	0.43 (0.22)	17.92 (±33.82)	0.27 (0.31)	8.43 (±31.71)	0.58 (0.15)
MPP [set 2] (W)	521.09 ± 98.87	532.53 ± 102.09	501.25 ± 83.04	−11.44 (±41.70)	0.56 (0.16)	19.84 (±3 1.58)	0.20 (0.36)	31.28 (±36.39)	0.09 (0.50)
MPP [set 3] (W)	501.46 ± 96.06	523.07 ± 107.75	492.48 ± 91.46	−21.61 (±42.53)	0.29 (0.29)	8.99 (±17.00)	0.27 (0.31)	30.59 (±33.19)	0.07 (0.53)
MPPmin [set1] (W)	430.41 ± 85.19	428.86 ± 99.11	424.17 ± 94.14	1.56 (±42.27)	0.94 (0.02)	6.24 (±34.24)	0.70 (0.11)	4.69 (±33.51)	0.77 (0.08)
MPPmin [set 2] (W)	425.78 ± 82.25	440.29 ± 90.33	391.97 ± 91.33	−14.51 (±51.24)	0.45 (0.21)	33.81 (±33.38)	0.02 * (0.74)	48.32 (±54.31)	0.03 * (0.65)
MPPmin [set 3] (W)	398.86 ± 88.74	422.53 ± 111.28	406.07 ± 99.94	−23.66 (±43.26)	0.29 (0.32)	−7.21 (±24.25)	0.53 (0.17)	16.46 (±46.57)	0.46 (0.20)
Work [set 1] (J)	2405.81 ± 532.92	2322.21 ± 493.88	2385.81 ± 549.18	83.59 (±107.05)	0.12 (0.45)	19.99 (±159.89)	0.79 (0.07)	−63.60 (±163.90)	0.42 (0.22)
Work [set 2] (J)	2388.63 ± 488.98	2342.16 ± 506.69	2377.38 ± 490.31	46.47 (±87.76)	0.27 (0.31)	11.25 (±96.19)	0.80 (0.07)	−35.22 (±94.29)	0.43 (0.22)
Work [set 3] (J)	2361.30 ± 502.87	2319.56 ± 504.58	2368.65 ± 493.40	41.74 (±77.47)	0.27 (0.31)	−7.35 (±116.55)	0.89 (0.04)	−49.09 (±133.67)	0.44 (0.21)
Work [total] (J)	7155.73 ± 1517.62	6983.94 ± 1489.85	7131.84 ± 1509.67	171.79 (±200.29)	0.09 (0.50)	23.89 (±338.94)	0.88 (0.04)	−147.90 (±328.57)	0.35 (0.26)

* *p* ≤ 0.05.

**Table 4:** Mean values ± standard deviation of the maximal value of mean propulsive velocity (MPV), minimal MPV value, and velocity loss (VL) in each set or training (total) in bench-press exercises. Differences and confidence intervals (95% CI), effect sizes (ES), and *p*-values are also presented.

	WU	WU80	WU40	WU vs. WU80		WU vs. WU40		WU80 vs. WU40	
				Difference (±95% CI)	*p*-Value (ES)	Difference (±95% CI)	*p*-Value (ES)	Difference (±95% CI)	*p*-Value ES
MPV [set 1] (m.s^−1^)	0.53 ± 0.07	0.52 ± 0.11	0.50 ± 0.10	0.01 (±0.05)	0.74 (0.09)	0.02 (±0.04)	0.15 (0.35)	0.02 (±0.04)	0.44 (0.19)
MPV [set 2] (m.s^−1^)	0.48 ± 0.09	0.48 ± 0.10	0.48 ± 0.08	0.00 (±0.06)	0.97 (<0.01)	0.00 (±0.03)	0.98 (<0.01)	0.00 (±0.04)	0.94 (<0.01)
MPV [set 3] (m.s^−1^)	0.50 ± 0.08	0.47 ± 0.11	0.47 ± 0.10	0.03 (±0.05)	0.18 (0.26)	0.03 (±0.03)	0.09 (0.40)	0.00 (±0.04)	0.89 (<0.01)
MPVmin [set 1] (m.s^−1^)	0.37 ± 0.11	0.36 ± 0.11	0.36 ± 0.09	0.01 (±0.06)	0.74 (0.07)	0.01 (±0.04)	0.73 (0.09)	0.00 (±0.04)	0.94 (<0.01)
MPVmin [set 2] (m.s^−1^)	0.33 ± 0.11	0.32 ± 0.13	0.33 ± 0.11	0.01 (±0.08)	0.85 (0.05)	0.00 (±0.04)	0.91 (<0.01)	−0.01 (±0.05)	0.70 (0.08)
MPVmin [set 3] (m.s^−1^)	0.32 ± 0.12	0.31 ± 0.12	0.31 ± 0.11	0.01 (±0.06)	0.61 (0.07)	0.01 (±0.05)	0.53 (0.09)	0.00 (±0.05)	0.99 (<0.01)
VL [set 1] (%)	30.65 ± 13.83	30.96 ± 12.23	28.19 ± 9.32	−0.31 (±6.33)	0.92 (0.02)	2.46 (±6.74)	0.46 (0.15)	2.77 (±5.17)	0.28 (0.22)
VL [set 2] (%)	33.42 ± 14.35	35.69 ± 16.73	32.81 ± 13.43	−2.27 (±9.87)	0.64 (0.09)	0.62 (±6.05)	0.84 (0.04)	2.89 (±6.81)	0.39 (0.17)
VL [set 3] (%)	35.96 ± 15.76	35.35 ± 13.66	35.54 ± 14.48	0.62 (±7.23)	0.86 (0.03)	1.42 (±7.90)	0.71 (0.02)	0.81 (±7.06)	0.82 (0.01)
VL [total] (%)	44.86 ± 12.98	43.81 ± 13.80	45.12 ± 13.11	1.04 (±6.79)	0.76 (0.06)	−0.27 (±5.78)	0.92 (0.02)	−1.31 (±5.34)	0.62 (0.10)

**Table 5:** Mean values ± standard deviation of the peak velocity (PV) and time to reach peak velocity (time to PV) in each set or training (total) in bench-press exercises. Differences and confidence intervals (95%CI), effect sizes (ES), and *p*-values are also presented.

	WU	WU80	WU40	WU vs. WU80		WU vs. WU40		WU80 vs. WU40	
				Difference (±95% CI)	*p*-Value (ES)	Difference (±95% CI)	*p*-Value (ES)	Difference (±95% CI)	*p*-Value ES
PV [total] (m.s^−1^)	0.82 ± 0.11	0.80 ± 0.15	0.82 ± 0.12	0.02 (±0.05)	0.43 (0.17)	0.00 (±0.04)	0.95 (<0.01)	−0.02 (±0.05)	0.45 (0.17)
Time to PV [set1] (ms)	574.77 ± 233.46	588.62 ± 334.46	694.50 ± 211.71	−13.85 (±137.71)	0.84 (0.04)	−119.73 (±70.36)	<0.01 ** (0.69)	−105.89 (±144.22)	0.14 (0.30)
Time to PV [set2] (ms)	533.19 ± 272.22	549.58 ± 293.34	662.31 ± 257.51	−16.39 (±96.37)	0.73 (0.07)	−129.12 (±120.81)	0.04 * (0.43)	−112.73 (±128.65)	0.08 (0.35)
Time to PV [set3] (ms)	583.19 ± 272.04	609.19 ± 334.18	575.61 ± 303.09	−26.00 (±119.23)	0.66 (0.09)	7.58 (±130.52)	0.91 (0.02)	33.58 (±171.65)	0.69 (0.08)

** *p* ≤ 0.01; * *p* ≤ 0.05.

**Table 6:** Mean values ± standard deviation of the maximal value of the mean propulsive power (MPP), the minimal value (MPPmin), and work developed in each set and total training (total) in bench-press exercises. Differences and confidence intervals (95% CI), effect sizes (ES), and *p*-values are also presented.

	WU	WU80	WU40	WU vs. WU80		WU vs. WU40		WU80 vs. WU40	
				Difference (±95% CI)	*p*-Value (ES)	Difference (±95% CI)	*p*-Value (ES)	Difference (±95% CI)	*p*-Value ES
MPP [set 1] (W)	309.30 ± 89.35	303.68 ± 87.97	292.25 ± 77.90	5.61 (±30.05)	0.70 (0.08)	17.04 (±20.09)	0.09 (0.34)	11.43 (±25.60)	0.37 (0.18)
MPP [set 2] (W)	288.13 ± 103.95	283.09 ± 82.62	286.45 ± 93.54	5.04 (±29.87)	0.73 (0.07)	1.68 (±18.47)	0.85 (0.04)	−3.35 (±22.28)	0.76 (0.06)
MPP [set 3] (W)	293.65 ± 97.98	279.92 ± 86.21	277.43 ± 98.77	13.74 (±47.49)	0.56 (0.12)	16.22 (±18.07)	0.08 (0.36)	2.48 (±48.18)	0.92 (0.02)
MPPmin [set 1] (W)	213.86 ± 79.99	211.38 ± 83.48	209.95 ± 66.28	2.48 (±33.78)	0.88 (0.03)	3.91 (±25.34)	0.75 (0.06)	1.43 (±26.28)	0.91 (0.02)
MPPmin [set 2] (W)	195.27 ± 94.72	184.76 ± 81.72	194.44 ± 85.96	10.51 (±42.15)	0.61 (0.10)	0.83 (±24.40)	0.95 (0.01)	−9.68 (±30.93)	0.53 (0.13)
MPPmin [set 3] (W)	184.26 ± 58.27	179.75 ± 79.56	183.65 ± 94.00	4.50 (±32.70)	0.78 (0.06)	0.61 (±29.79)	0.97 (0.01)	−3.89 (±32.57)	0.81 (0.05)
Work [set 1] (J)	1600.42 ± 448.60	1587.83 ± 427.57	1555.32 ± 440.22	12.59 (±44.22)	0.56 (0.12)	45.10 (±34.99)	0.01 ** (0.52)	32.51 (±37.61)	0.09 (0.35)
Work [set 2] (J)	1580.15 ± 453.53	1550.15 ± 421.00	1542.10 ± 461.30	30.00 (±43.32)	0.16 (0.28)	38.05 (±30.84)	0.02 * (0.49)	8.04 (±40.15)	0.68 (0.08)
Work [set 3] (J)	1569.33 ± 418.91	1534.35 ± 417.90	1534.38 ± 456.36	34.98 (±57.36)	0.22 (0.25)	34.95 (±41.02)	0.09 (0.35)	−0.03 (±45.16)	0.99 (<0.01)
Work [total] (J)	4749.90 ± 1312.99	4672.32 ± 1261.93	4631.80 ± 1355.01	77.59 (±125.19)	0.21 (0.25)	118.11 (±87.65)	0.01 ** (0.54)	40.52 (±111.54)	0.46 (0.15)

** *p* ≤ 0.01; * *p* ≤ 0.05.
